# Report of a Case of Chronic Endartaritis

**Published:** 1886-08

**Authors:** Simeon Tucker Clark

**Affiliations:** Professor of Medical Jurisprudence in the Medical Department of Niagara University


### *REPORT OF A CASE OF CHRONIC ENDARTARITIS.*

By SIMEON TUCKER CLARK, A. M., M. D.,
Professor of Medical Jurisprudence in the Medical Department of Niagara University.

About 9 o'clock, P. M., June 2, 1886, I was called to see Miss H., aged 33 years, and found she had just rallied from a fainting-fit, so complete as to render her insensible for five minutes. This attack had been preceded by intense pain in the right arm and hand; continued the entire day. She very properly attributed her syncope to this agonizing pain, which was still almost unendurable, and did not completely yield to large and repeated doses of morphia.

On the morning of the 3d, the next day, I found the right fore-arm cold and discolored; the right hand without sensation or motion. There was no pulsation of the radial artery.

The next day, June 4th, the hand was mummified and the fore-arm in a state of acute gangrene, the line of demarcation being sharply defined.

From Miss H., and her father, I obtained the following history: At the age of four years and upwards, calcareous concretions began to be deposited in the joints of the phalanges; and, when about fourteen years old, the right wrist and elbow-joints were almost anchylosed; the little finger of that hand was but partially developed, and remained so.

Miss H. had never been of robust health, but, for the most part of the time, did not complain.

The past winter she suffered much from neuralgia, mostly confined to the right side, but occasionally had brief attacks of *angina pectoris*, always attended with faintness and difficulty of breathing. The last four menstrual periods had been attended with great distress, and all the blood discharged was in clotted masses; in some instances, from her description, complete casts of the uterine cavity were expelled, red in color, and of the consistency of modeling-clay. At these periods, she described her sensations as peculiar; loss of vision, of short duration, but frequently repeated; partial aphasia, vertigo and loss of co-ordination in the right arm and fingers, palpitation, and not unfrequently a sluggish movement of the heart. For the last two months, had a feeling as if her head was of wood, or not a part of herself, and had lost consciousness for a moment on several occasions. Had not been warm this spring, and suffered greatly from cold all last winter.

Careful examination failed to furnish any physical signs of organic change of the heart or aorta, but feebleness of cardiac impulse and retarded pulse-wave were observed.

On the 5th, Dr. Wm. B. Gould was called in consultation, who verified my diagnosis of acute gangrene from endartaritis, and informed Miss H. that she would be forced to lose her right arm to preserve her life.

June 7th, Prof. W. S. Tremaine, M. D., of Buffalo, was called to see the case, and gave us the benefit of his large and varied surgical experience and ripe judgment. He advised early amputation, but gave an unfavorable prognosis. On the afternoon of the 9th, I amputated the right arm at the junction of the middle with the upper third, making a circular operation. The tissues at this point were warm and of normal color, although the brachial artery did not pulsate; in fact, there was little need of a ligature. Drs. Gould, Foote and Outwater, who assisted me, hoped sufficient blood supply would be obtained from the posterior circumflex and subscapularis arteries to enable the stump to heal. The night after the amputation, was the most comfortable she had passed since the acute invasion.

The evening of June 10th, or twenty-four hours after the operation, I observed that pulsation had ceased in the right subclavian artery, and my patient was very restless. She now complained of general numbness and coldness of the right side, and occasional cramps in the fingers of the left hand.

At 9 A. M., June 11th, pulsation was almost wanting in the left brachial artery, and the left fore-arm was becoming mottled. Temperature, 105°, restlessness increased, and thirst tormenting. This condition advancing, in spite of liberal nourishment and stimulation, gave little hope of re-action. About 4 P. M., fluids swallowed provoked nausea, and the feet became cold, although artificial heat, both wet and dry, was persistently applied. At 6 P. M., death occurred. Intelligence was perfect to the end.

This case presents many points of interest, but is sadly deficient in our failure to obtain an autopsy. The amputated member was carefully dissected. Much to my surprise, the stiffness of the elbow-joint was not due to calcareous or ossific deposits, but to products of inflammation—bands of adventitious tissue crossing each other in every direction, seeming, in the almost complete destruction of the anatomy of the parts, to be the products of a fatty degeneration, the sequellæ of an acute inflammation.

The *radial* artery was so changed, as to be with difficulty distinguished from the radial nerve, which it closely resembled, its lumen being obliterated and having become a white lusterless cord.

The deep palmer arch was destroyed, except a portion of the *superficialis-volæ*, which was in a state of acute inflammation, and served to connect the two arches in an imperfect manner.

The *ulnar* artery was filled with a substance resembling brick-dust, finely powdered, mixed with oil and closely packed; this substance was identical in appearance with that which filled the *brachial* artery. All those arteries which I was permitted to examine, presented no change of the internal coatings, if we except unusual redness. There were no superficial elevations or atheromatous patches. On the contrary, the external coats were inflamed, thickened, opaque, and in some parts sclerosed; and, although their canals did not seem lessened in diameter, they were completely plugged throughout their whole extent. Of course, this does not refer to such vessels as had been for years passing into a state of complete degeneration, but such as had more recently performed their functions.

The superficial palmer arch was still in form, but impervious, save a small part of the *volæ*, by which, as before stated, the two arches had maintained a quasi connection.

By these pathological conditions, we are reminded of the teachings of the renowned Meigs, who ever insisted that "the normal quality of blood depended upon the integrity of the intima of veins and arteries." "The endangium contains the force that makes blood." "Contact with the endangium is essential to the development of blood originally, since it loses its physical character as soon as it ceases from that contact, or the character of the blood-making intima is demoralized."

"The endangium is the blood-membrane. When it is healthy, the blood is so; when it is diseased, the blood becomes something more or less than healthy blood." "The health of the endangium is as essential to a normal hæmatosis as that of the gastro-intestinal mucous membrane is to the health of the digestive force."

Rokitansky, many years ago, gave it as his opinion that in these cases a deposit of blood took place, but, after the observations of Virchow, Traube and others concerning the microscopic changes which were found to be constant in the intima, he renounced his original hypothesis, and concluded the whole affection to be inflammatory.

It seems to us that in this case we have not only a chronic, but acute and progressive, endartaritis, and that a change of the intima of the blood-vessels must in every case result in a change of the blood itself, not only in the affected vessels, but the entire circulation. This is confirmation of the united doctrines taught by Meigs, Rokitansky, Traube, and noticeably Barié, who has written learnedly of acute arteritis following typhoid or enteric fever, and who pronounces "the two factors in this disease to be local and permanent irritation by parasitic and infectious germs, and profound disturbance of the vaso-motor nerve supply from whatever source." All of these authors have given us light.

This case proves to our mind that, in endartaritis, change takes place primarily in the intima of the vessels; and after the blood-making membrane becomes somewhat generally affected, the blood itself becomes a changed fluid, tending to deposits, clots, thrombi, pigmentary immigrations, and even exudates of adventitious products.

